# Interferon beta drives therapy resistance in a patient‐derived model of high‐grade serous ovarian cancer

**DOI:** 10.1002/1878-0261.70305

**Published:** 2026-07-10

**Authors:** Ashlyn Conant, Tise Suzuki, Kiera McGivney, V. S. S. Abhinav Ayyadevara, Sharon Asariah, Jay Deng, Ethan Nyein, Jacqueline Coats, Gary Yu, Yevgeniya J. Ioffe, Christian Hurtz, Juli J. Unternaehrer

**Affiliations:** ^1^ Division of Biochemistry, Department of Basic Sciences Loma Linda University California USA; ^2^ Department of Biochemistry Whitworth University Spokane Washington USA; ^3^ Division of Cancer Sciences, Department of Basic Sciences Loma Linda University California USA; ^4^ Department of Biology University of Redlands California USA; ^5^ Department of Gynecology and Obstetrics Loma Linda University California USA; ^6^ Division of Gynecologic Oncology, Department of Gynecology and Obstetrics Loma Linda University California USA; ^7^ Present address: Department of Biology/Allied Health Southern Adventist University Collegedale Tennessee USA; ^8^ Present address: School of Medicine California University of Science and Medicine Colton California USA

**Keywords:** cisplatin resistance, DNA‐damage, interferon‐beta, JAK/STAT signaling, ovarian cancer

## Abstract

Type 1 interferon (IFN‐1) production and signaling are frequently activated in response to DNA damage and have been associated with the development of therapy resistance in cancer. However, the cell‐autonomous role of IFN‐1 in driving resistance in high‐grade serous ovarian cancer (HGSOC) remains unclear. Specifically, whether IFN‐1 functions in HGSOC as solely a response to genotoxic stress due to genotoxic therapy as frontline treatment, or can independently act in driving resistance phenotypes, has not been studied. Utilizing a patient‐derived model of cisplatin‐sensitive (SE) and ‐resistant (CR) HGSOC, we demonstrate that chronic cisplatin exposure is associated with enrichment of IFN‐1 signaling and an interferon‐related DNA damage resistance signature. Acute cisplatin treatment elicited dynamic IFN‐1 signaling in both SE and CR cells, indicating a conserved stress response. However, chronic, low‐level exposure to exogenous IFNβ, without a DNA‐damaging agent, phenocopied several features of chronic cisplatin‐driven resistance, including reduced therapeutic sensitivity and decreased proliferation. Together, these findings identify IFNβ as a driver of resistance‐associated phenotypes and highlight cell‐autonomous IFN‐1 signaling as a potential biomarker for resistance and therapeutic target in platinum‐resistant disease.

## Graphical abstract

Interferon type 1 (IFN‐1) production and signaling is associated with the acquisition of therapy resistance, following chronic DNA damage, via Interferon‐related DNA damage resistance signature (IRDS) gene expression. An alternative, DNA damage‐independent role of sustained IFN‐1 mediated resistance was identified and characterized by the emergence of a slow‐cycling, drug‐tolerant phenotype. Created in BioRender. Conant, A. (2026) https://BioRender.com/1qrch8v.

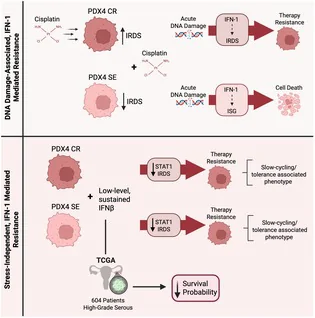

**Abbreviations**
CR: resistant
HGSOC: high‐grade serous ovarian cancer
IFN: interferon
IFN‐1: interferon type 1
IFNα: interferon Alpha
IFNβ: interferon Beta
IRDS: interferon‐related DNA damage resistance signature
ISG: interferon‐stimulated gene
ISGF3: interferon‐stimulated gene factor 3
PDX: patient‐derived xenograft
SE: sensitive
TCGA: The Cancer Genome Atlas
u‐ISGF3: unphosphorylated Interferon‐stimulated gene factor 3

## 1 Introduction

High‐grade serous ovarian carcinoma (HGSOC) remains one of the deadliest gynecologic malignancies despite a more than 80% response rate to frontline therapy [1, 2]. Standard of care consists of surgical debulking followed by 6 cycles of platinum/taxane adjuvant chemotherapy [3]. For patients who are not primary debulking candidates, neoadjuvant chemotherapy, followed by interval debulking surgery (IDS) and adjuvant therapy, are utilized. Availability of targeted therapies has been steadily increasing but remains limited; vascular endothelial growth factor (VEGF) inhibitors, such as bevacizumab, and poly‐ADP ribose polymerase (PARP) inhibitors, such as olaparib, and antibody drug conjugates (ADCs) such as mirvetuximab, being some of the most common therapies for women with specific indications [3, 4, 5]. Regardless of the robust frontline response, more than 70% of women experience recurrence and associated therapy resistance, contributing to a bleak 30% 5‐year survival rate for stage IV disease [6]. The initial responsiveness coupled with subsequent treatment‐resistant recurrence combine to demonstrate the urgent need for reliable models of acquired therapy resistance.

Within tumor cells, genomic instability, DNA damage, and aberrant mitotic events all contribute to the accumulation of cytosolic DNA, which activates the cGAS‐STING (cyclic GMP‐AMP synthase—stimulator of interferon genes) pathway and promotes type 1 interferon (IFN‐1) production [7, 8, 9, 10]. This mechanism is also amplified by DNA‐damaging therapies, such as chemotherapy, and by the presence of damage‐associated molecular patterns (DAMPs) in the tumor microenvironment (TME) [11, 12].

The intensity and duration of the IFN‐1 signal greatly influence the cellular response and phenotypic adaptation via interferon‐stimulated gene (ISG) induction [13]. Canonical ISG expression is driven by a robust and acute IFN‐1 signaling event, which is transduced via canonical JAK/STAT signaling, leading to the formation and phosphorylation of the interferon‐stimulated gene factor 3 (ISGF3) transcription factor complex. This signaling correlates with antiproliferative, pro‐inflammatory, pro‐apoptotic, cytotoxic phenotypes [14, 15]. Noncanonical ISG signaling, driven by sustained STING activation and chronic low‐level IFN‐1 production, drives noncanonical JAK/STAT signaling and formation of the unphosphorylated ISGF3 (u‐ISGF3) complex. This noncanonical signaling promotes a protumorigenic, immunosuppressive, and anti‐apoptotic phenotype that results in cytoprotection [16]. A subset of ISGs termed the interferon‐related DNA damage resistance signature (IRDS) is highly associated with resistance to DNA damaging therapies [17, 18]. This gene set is a pathologically sustained response to noncanonical IFN‐1 signaling and has been found to be present, and potentially predictive, in ovarian cancer as early as detection of a p53 mutation in the fallopian tube [19, 20]. However, it remains unclear whether IFN‐1 signaling in HGSOC is merely a consequence of DNA damage or whether it can independently drive therapy resistance and phenotypic adaptation.

In this study, we aimed to define the role of cancer cell autonomous IFN‐1 production and signaling in the development of therapy resistance and associated phenotypes within HGSOC. Using a syngeneic patient‐derived model of cisplatin‐sensitive and ‐resistant HSGOC, we demonstrate that chronic cisplatin exposure is associated with enrichment of IFN‐1 and IRDS signatures. Acute cisplatin treatment is shown to differentially and temporally enhance IFN‐1 production, as well as related signaling in sensitive and resistant cells, highlighting the effect of prior therapy on stress response. In validation of the role of DNA damage‐induced IFN‐1 activation, sensitive and resistant cells treated with chronic, low‐level IFNβ phenocopied key features of cisplatin resistance, including altered cell states and reduced cisplatin sensitivity. Notably, IFNβ‐driven resistance occurred in the absence of sustained IRDS or canonical ISG induction, indicating the existence of alternative mechanisms by which IFN‐1 promotes therapeutic response adaptation. These findings identify IFNβ as a functional driver of cisplatin resistance and associated phenotypes, highlighting cell‐autonomous IFN‐1 signaling as a potential biomarker and therapeutic target.

## 2 Materials and methods

### 2.1 Cell culture

The collection and use of the cells in this study were approved by the Loma Linda University (LLU) Institutional Review Board (IRB, 58328) according to the standards set by the Declaration of Helsinki. The experiments were undertaken with the understanding and written consent of each subject. Participants gave written and informed consent. Deidentified tumor tissue was collected by the Loma Linda University Cancer Center Biospecimen Laboratory (LLUCCBL) and immediately transported to the laboratory for processing. PDX samples were collected, preserved, and processed as previously described [21]. The same tumor samples were used to clinically diagnose HGSOC.

Patient‐derived cells (PDX4 SE/CR, PDX6, PDX1) were cultured in a 3 : 1 mixture of HycloneTM Ham's Nutrient Mixture F12 with L‐glutamine (SH30026.01; Cytiva) and Dulbecco's Modified Eagle's Medium with high glucose and L‐glutamine (DMEM; 25–501), supplemented with 5% FBS (Omega Scientific), 0.4 μg·mL^−1^ hydrocortisone (H0888‐1G; Sigma‐Aldrich), 5 μg·mL^−1^ insulin (91077C‐100MG; Sigma‐Aldrich), 2 μg·mL^−1^ isoprenaline hydrochloride (I5627‐5G; Sigma‐Aldrich), 24 μg·mL^−1^ adenine (A8626; Sigma‐Aldrich), 100 U penicillin, and 100 μg/mL streptomycin (25–512; Genesee Scientific). The purity and identity of PDX4 were confirmed before and after the generation of cisplatin resistance utilizing Short Tandem Repeat validation (2024) (Laragen Inc., Culver City, CA, USA).

OVCAR8 cells (RRID:CVCL_1629), a kind gift of Dr. Carlotta Glackin (City of Hope, Duarte, CA, USA), were cultured in Dulbecco's Modified Eagle's Medium with high glucose and L‐glutamine (DMEM; 25–501; Genesee Scientific), supplemented with 10% FBS (Omega Scientific) and 100 U penicillin and 100 μg·mL^−1^ streptomycin (25–512; Genesee Scientific).

Immortalized fallopian tube secretory epithelial cells (FTSEC 240), a generous gift of Dr. Ronny Drapkin [22] (University of Pennsylvania, Philadelphia, PA, USA), were grown in HycloneTM Dulbecco's Modified Eagle's Medium/F12 (SH3002302, Fisher Scientific), Ultroser G serum substitute (67042.1/s, Crescent Chemical Company), and 100 U penicillin, and 100 μg·mL^−1^ streptomycin (25–512; Genesee Scientific).

Cells were treated with their respective IC50's of cis‐diammineplatinum(II) dichloride (cisplatin; D3371‐100MG; TCI Chemicals) based on cell viability assays [21, 23].

Cells were treated with Human Interferon Beta 1a at 100 U·mL^−1^ or 2 U·mL^−1^ (11415–1; PBL Assay Science) diluted in 0.1% BSA in sterile 1× PBS.

Cell authentication was done via short tandem repeat validation (Laragen Inc., Culver City, CA, USA) within the past three years, before and after the generation of cisplatin resistance. All experiments were conducted using mycoplasma‐free cells. Cells were routinely tested using the PlasmoTestTM Mycoplasma Detection Kit (rep‐pt1; InvivoGen, San Diego, CA, USA).

### 2.2 Library preparation and RNA sequencing

Total RNA was extracted from PDX4 SE and PDX4 CR cells using the miRNeasy Mini Kit (217 004, Qiagen, Germantown, MD, USA). Subsequently, RNA‐seq library construction and generation of raw data was performed at the Loma Linda University Center for Genomics using the Ovation Universal RNA‐seq System (0364; Tecan; Männedorf, Switzerland). Briefly, 100 ng of total RNA was reverse transcribed and then made into double‐stranded cDNA by adding a DNA polymerase. cDNA was concentrated using Agencourt beads, followed by end repair and adaptor ligation. Unique barcodes were used for each sample for multiplexing. Targeted rRNA‐depletion was performed before the final library construction. Libraries were amplified using 13 cycles in the Eppendorf™ Mastercycler™ pro PCR system (Hamburg, Germany) and purified using Agencourt beads. RNA‐seq libraries were sequenced on Illumina NextSeq 550 (Illumina, San Diego, CA, USA) with single 76‐bp reads. The Illumina RTA v 2.4.11 software was used for basecalling, and bcl2fastq v 2.17.14 was used for generating FASTQ files.

Specific analysis pipelines and codes used to generate graphs can be found at https://github.com/tsuzukiPhD/RNAseq (accessed on 8 January 2024), and bioinformatics analysis was done as described [23]. Briefly, FASTQC (v 0.11.9) and MultiQC (v 1.11) were used to determine sample quality before and after trimming reads with Trimmomatic (v 0.39), after which STAR (v 2.7.10a) was used to align to the human genome using GRCh38 (accessed on 4 May 2023), release 109, as reference. SAMtools (v 1.12) were used for sorting and indexing binary alignment files. HTSeq (v 2.0.2) was used to assemble RNA‐seq reads into transcripts, counting transcripts, and for producing a count matrix for the differential gene expression analysis between samples; DESeq2 (v 1.43.1) was used for this purpose. To enhance robustness of gene expression analysis, genes with less than 10 counts were excluded from matrix. Ensembl identifiers were converted to HGNC symbols using BioMart R package (v 2.58.0).

### 2.3 Pathway analysis

Ingenuity Pathway Analysis (IPA; QIAGEN Inc., Hilden, Germany, https://digitalinsights.qiagen.com/IPA; accessed on 5 May 2023) was used for the identification of canonical/hallmark pathways activated among the DEGs obtained through the PDX4 RNA‐seq analysis [24]. To identify specific activated pathways associated with interferon signaling and JAK/STAT, the following keywords were used for filtering: ‘interferon’ and ‘JAK’. Bar charts were created with GraphPad Prism v 10.2.2.

Gene Set Enrichment Analysis (GSEA; v 4.3.2; accessed on 27 October 2023) was also used for the identification of pathways activated in the DEGs of PDX4 CR [25, 26]. Both Hallmark Gene Sets and Gene Ontology (GO): Biological Processes pathways were queried. Since gene set permutation was used, only pathways with an FDR of less than 0.05 were considered for further analysis. Permutations for GO were set at 1000, and Hallmark Gene Sets at 50 000. Dot plots were created with ClusterProfiler [27] and display the results of a Gene Ontology (GO) enrichment analysis for the “activated” and “suppressed” DEGs.

### 2.4 Quantitative real‐time polymerase chain reaction (RT‐qPCR)

RNA from cells was isolated using the IBI Isolate DNA/RNA Reagent Kit (IB47602, IBI Scientific, Dubuque, IA, USA) according to the manufacturer's recommendations. cDNA was synthesized from either 500 ng or 1 μg of total isolated RNA using the Maxima First Strand cDNA Synthesis Kit (K1672; Thermo Fisher Scientific). RT‐qPCR was performed using Applied Biosystems™ PowerUP™ SYBR™ Green Master Mix (A25778; Thermo Fisher Scientific) and gene primers (Methods, Table S1; custom ordered as cDNA oligos from IDT) on a Stratagene Mx3005P Instrument (Agilent Technology, Santa Clara, CA, USA). The results were analyzed using the Δ cycles to threshold (ΔCt) method.

### 2.5 IFN‐α/β protein detection

Secreted IFNα/β was detected using an interferon stimulated gene factor 3 (ISGF3)–secreted alkaline phosphatase (SEAP) HEK‐Blue IFNα/β reporter cell line (hkb‐ifnabv2; InvivoGen). Concentrations of IFNα/β were calculated by measuring absorbance at 620 nm on a Biotek Synergy H1 Multimode plate reader (Agilent Technology, Santa Clara, CA, USA). A standard curve was generated using a twofold serial dilution of IFN‐β starting at 50 U·mL^−1^ and plotted with hyperbolic non‐linear regression on GraphPad Prism v10.5.0.

### 2.6 Cell viability assay

Cell viability assays were completed using thiazolyl blue tetrazolium bromide (MTT; 00697; Chem‐Impex) assays as previously described [21, 23].

### 2.7 Flow cytometry

Permeabilized flow cytometry was completed as follows. Cells were stained with BD Horizon™ fixable viability stain (FVS) 780 (565 388; BD) according to the manufacturer's recommendations for compensation and live cell gating. A single heat shocked sample was used as a positive gating control for FVS. Cells were then permeabilized using 90% ice‐cold methanol for 1 h. Samples were brought to room temperature before rinsing 3× with FACS stain [1% FBS, 0.1% sodium azide (NaN_3_; s2002‐5 g; Sigma), and 2 mm EDTA in PBS]. Cells were labeled using two staining methods. Cells were stained with directly conjugated fluorescent dye antibodies against STAT1 AlexaFluor™ 647 (558 560; BD) and pSTAT1 PE (612 564; BD), incubated at room temperature for 30 min, then washed and resuspended in FACS stain. Cells were also stained using a two‐step approach first staining with IFITM1 (99969S; Cell Signaling Technology) diluted in 0.5% BSA for 1 h at room temperature before rinsing and staining with AlexaFluor™ 647 goat anti‐rabbit IgG (H + L) secondary antibody (A21245; Fisher Scientific) at room temperature for 30 min, washed, and resuspended in FACS stain. UltraComp eBeads (01–2222; Thermo Fisher Scientific), secondary only, and fluorescence minus one (FMO)‐stained samples were used for positive and negative gating controls where appropriate.

All flow cytometry was performed on MACSQuant Analyzer 10 (Miltenyi Biotec, Bergisch Gladbach, Germany), and data analysis was performed using FlowJo v10.10.0 (FlowJo LLC, Ashland, OR, USA).

### 2.8 Cell cycle analysis

PDX4 cells were treated with 10 ng·μL^−1^ IFN‐β for 72 h in 6‐well plates, followed by IFN‐β withdrawal by replacing the spent media with 2 mL of fresh culture media. Twenty‐four hours later, cells were treated with 10 μm BrdU (559 619; BD Biosciences) for 30 min, 4 h, or 18 h. Cells were then harvested using 0.05% Trypsin–EDTA (25200–056, Gibco), and samples were prepared for flow cytometric analysis following the manufacturer's recommended protocol.

### 2.9 Microscopy

Cell images were obtained using a Nikon Eclipse Ti microscope (Nikon Instruments, Melville, NY, USA) and micro-manager v1.4.22 software. Aspect ratio was calculated by measuring and dividing the width and length of 20 individual cells per photograph using ImageJ software (National Institutes of Health, Bethesda, MD, USA).

### 2.10 Proliferation assay

Cells were plated at 50 000 cells per well in a 12‐well plate. Individual wells were trypsinized at various timepoints up to 120 h and counted using a trypan blue exclusion cell counting method.

### 2.11 In silico analyses

Gene‐expression analyses from The Cancer Genome Atlas (TCGA: (https://portal.gdc.cancer.gov/)) was based on a defined gene set of the interferon/innate immunity pathway (IFNB, IFITM1, PLSCR1, OAS1, STAT1) to test our *a priori* working hypothesis and develop our composite molecular biomarker risk score. A survival analysis approach (Kaplan–Meier curves, univariate Cox proportional hazards model) was taken to analyze a genetic array of differential expression from ovarian cancer patients in TCGA, stratified by high vs. low expression of interferon/innate immunity. Box and whisker plots were visually expressed to examine the differences in the distribution of generic expression, stratified by treatment status for ovarian cancer (none versus prior therapy).

Kaplan–Meier: Survival analysis was conducted for time to death in days as a right‐censored survival outcome, stratified by high versus low expression of the interferon/innate immunity composite score using the median value as the cutoff. A log‐rank test was performed to assess for differences in the median (50th percentile) survival times between both Kaplan–Meier curves. The difference in the time in days gained at the median survival time is a primary clinical outcome for the impact of a higher composite score versus a lower composite score. Tick marks above the curves represent right‐censoring due to loss‐to‐follow‐up and the last available visit date was used to maximize the catamnestic information on survival, while each incremental step‐down in the curve represents a true event of death. Below the Kaplan–Meier stratified curves is the Number at risk actuarial table that provides the level of the precipitous drop in patients still remaining alive at each time point, which corresponds to the steepness of the survival curves.

Forest Plot—Cox Proportional Hazards TCGA Ovarian Cancer: A forest plot of the set of interferon/innate immunity genes [IFITM1, OAS1, PLSCR1, IFNB1, STAT1, and the composite score (average of the normalized z‐scores of the gene expression value of the set of 5 genes)] produced a stacked ordered comparison of the magnitude (highest to lowest) of the hazard ratio (HR) and its affiliated 95% confidence intervals {HR*exp(± 1.96*ln[SE(HR)])}. The plot was generated using the reciprocal values for the HR values (1/original HR values) and the new 95% CI (1/lower bound of the 95% CI; 1/upper bound of the 95% CI). The unique HR reported is the individual genetic risk factor independently associated with the hazard outcome in a univariate Cox proportional hazards model, where a HR > 1 corresponds to higher genetic expression associated with lower rates of survival or higher hazards of death.

Box Plot of Gene Expression by Prior Treatment [No (Blue) vs. Prior (Red)]: Among each gene, side‐by‐side box plots for no (blue) versus prior (red) ovarian cancer treatment status were visualized for the mathematically transformed outcome of the expression of log_2_(FPMK+1), where FPMK is the fragment per kilobase of transcript per million mapped reads. The box plots comprises of the middle bar inside of the box (median; 50% percentile), the lower box line (Q1; 25th percentile), the upper box line (Q3; 75th percentile), the lower whisker tail [Q1–1.5*IQR(inter‐quartile range;Q3‐Q1)], the upper whisker tail [Q3 + 1.5*IQR(inter‐quartile range;Q3‐Q1)] and the dots outside the whisker tails are the extreme outlier values. Wilcoxon Mann–Whitney nonparametric rank‐sum tests were performed to test for differences in the distribution in the none versus prior treatment groups, stratified by the specific genetic markers.

For *in silico* analyses, R scripts for data acquisition, preprocessing, differential expression analysis (DESeq2), and visualizations were developed with assistance from Claude (Anthropic, version 3.5 Sonnet, 2026, accessed on 25 March 2026). All analytical outputs and biological interpretations were independently reviewed and verified by the research team. The authors reviewed and edited the R script‐generated output and take full responsibility for the content of the publication.

### 2.12 Statistical analysis

For all experiments, samples in the same treatment group were harvested from at least three biological replicates and tested in three technical replicates. All values in the figures and text are the mean ± SD. Graphs were generated, and statistical analyses were performed using Prism v 10.5.0. Statistically significant differences were determined by unpaired *t*‐tests or paired *t*‐tests, as appropriate, unless otherwise noted. *P*‐values of less than 0.05 were considered significant. Outliers were not removed.

## 3 Results

### 3.1 Chronic cisplatin exposure induced an IFN‐1 associated resistant state

**Fig. 1:**
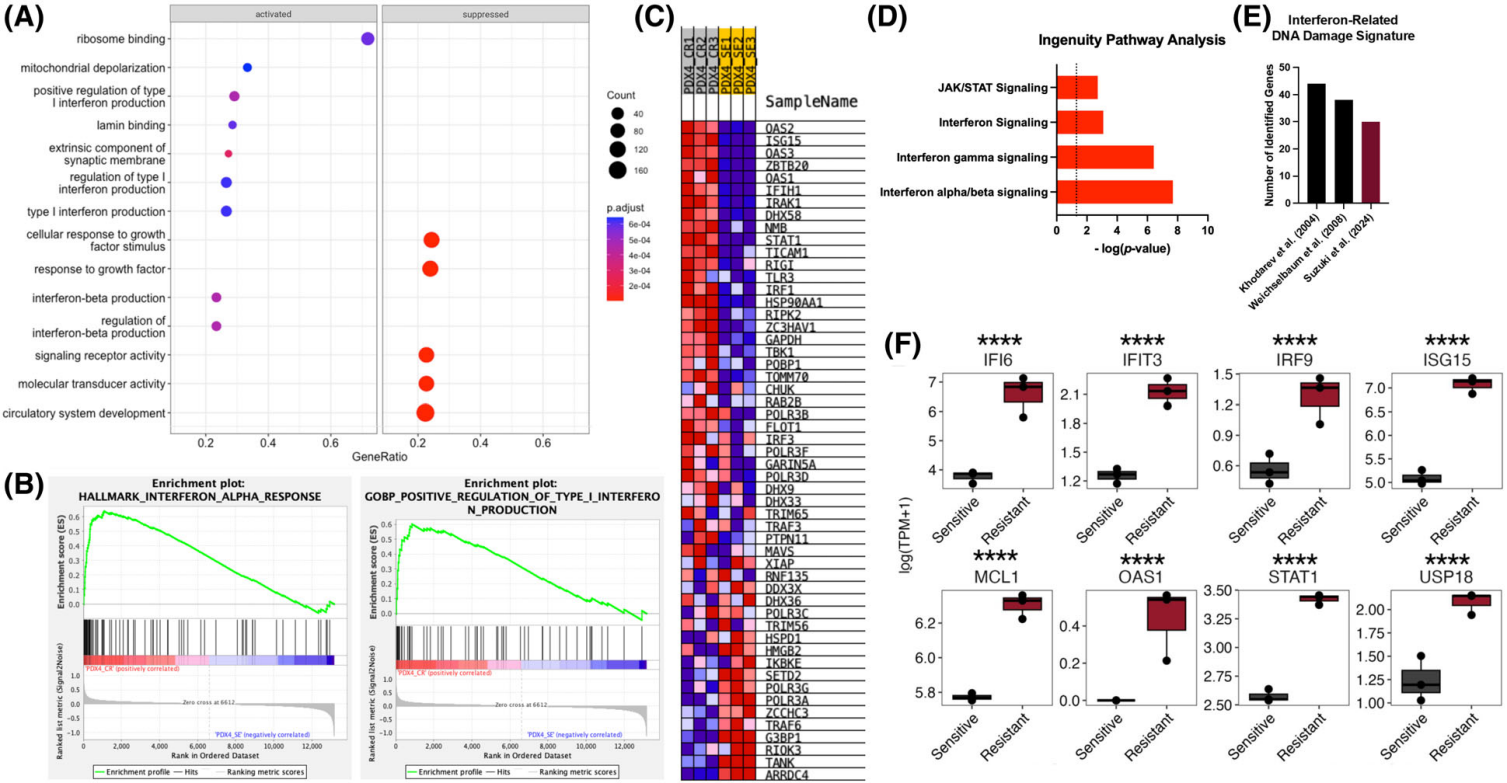
RNA Sequencing of cisplatin sensitive and resistant cells reveals IFN‐1 production and signaling signatures. (A) Gene ontology enrichment analysis of biologic processes activated and suppressed in PDX4 CR compared to SE. (B) Gene Set Enrichment Analysis (GSEA) of PDX4 cells reveals positive enrichment of the Hallmark Interferon Alpha Response and GO: BP‐Positive Regulation of Type 1 Interferon Production Pathways in PDX4 CR. (C) A GSEA Blue‐Pink O′ Gram in the Space of the Analyzed GeneSet histogram displaying the specific genes enriched within the “GO: BP‐Positive Regulation of Type I Interferon Production” pathway for the PDX4 samples. The gene expression values are represented in a range of colors, from dark red (upregulation) to dark blue (downregulation). The darker colors correspond to higher differential expression, while the lighter colors correspond to lower differential expression. (D) Ingenuity pathway analysis (IPA) displaying upregulation of IFN‐1‐related signaling pathways. (E) Comparison of the actual number of genes characterized as being involved in the interferon related DNA damage signature (IRDS) expressed in PDX4 CR versus those detailed in reference [17, 18]. (F) Gene expression profiles of IRDS genes in PDX4 SE (sensitive) and CR (resistant) [23]. Box and whisker plots show median (dark line), first and third quartile (boxes), minimum and maximum outlier values (black dots). Statistical significance was determined using the unpaired *t*‐test. *P*‐values: *P* ≤ 0.0001 (****).

In a previous study utilizing a syngeneic chemosensitive and resistant patient‐derived model of HGSOC, RNA sequencing revealed JAK/STAT among the most upregulated signaling pathways in a chemoresistant cell line (PDX4 CR) when compared to its sensitive counterpart (PDX4 SE) [23]. Gene ontology (GO) enrichment analysis of differentially regulated biological processes in CR compared to SE samples revealed significant activation of pathways involved in IFN‐1 production and signaling (Fig. 1A). Additionally, Gene Set Enrichment Analysis (GSEA) and Ingenuity Pathway Analysis (IPA) of the sequencing further indicated that IFN‐1 production/response gene sets, along with the JAK/STAT pathway, were significantly enriched among the differentially expressed genes (DEGs) of PDX4 CR (Fig. 1B–D). Analysis of individual sample replicates revealed that cisplatin‐resistant samples exhibited marked activation of specific genes associated with IFN‐1 production (Fig. 1C).

The Interferon‐Related DNA Damage Signature (IRDS) is a group of genes associated with chemo‐ and radio‐therapy resistance in several cancer cell types [17, 18]. Approximately 30 genes in the IRDS set were identified, compared to those reported in foundational IRDS publications [17, 18] (Fig. 1E). Several specific IRDS genes, such as IFI6, IFIT3, IRF9, ISG15, MCL1, OAS1, STAT1, and USP18, were significantly upregulated within the CR cells (Fig. 1F). These findings indicate a possible role for IFN‐1 signaling in the development of HGSOC chemoresistance.

### 3.2 IFN‐1 production and signaling are transcriptionally engaged, while cytokine output is limited

**Fig. 2:**
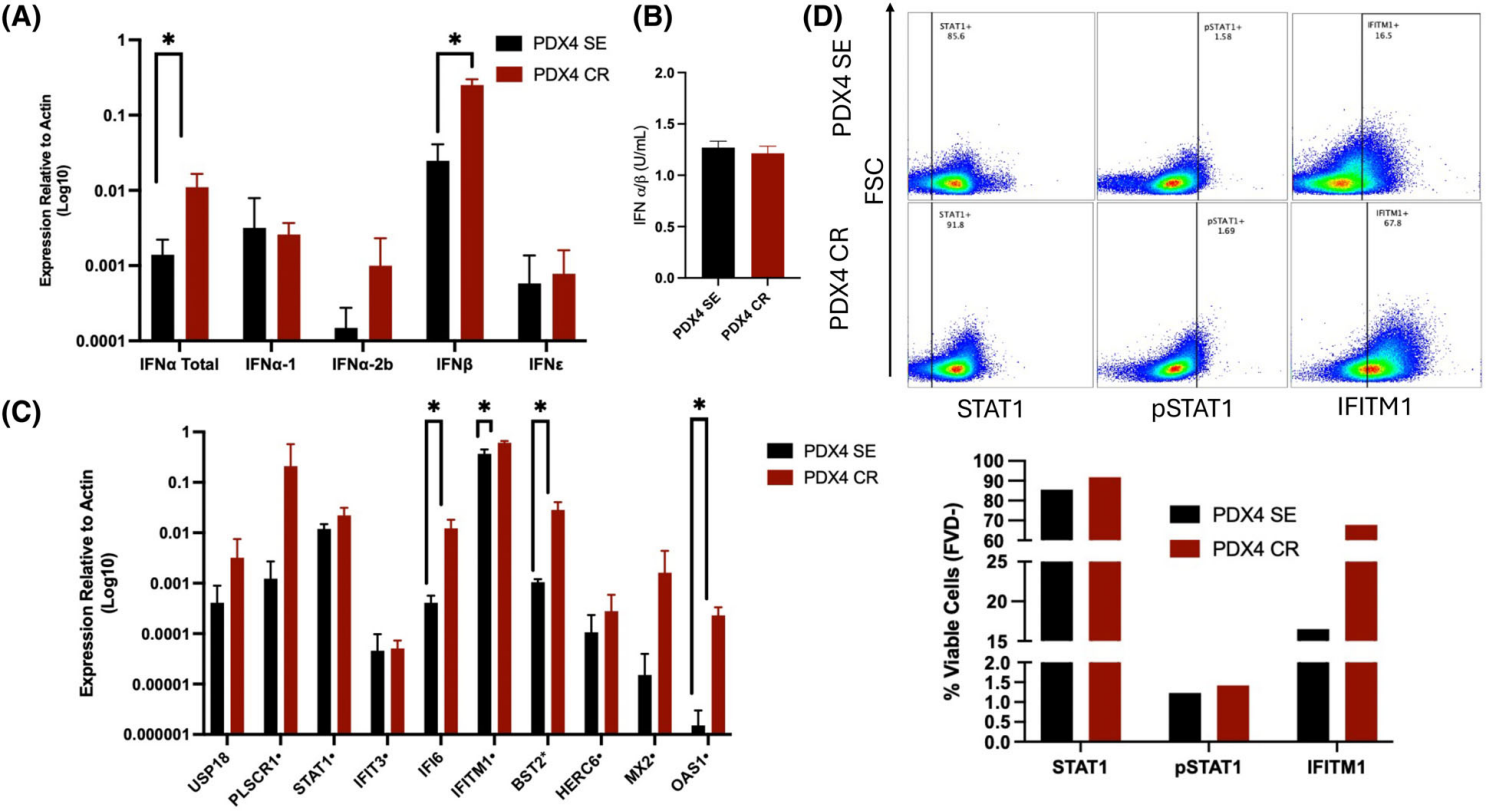
The IFN‐I pathway is activated in CR cells. (A) RT‐qPCR analysis of IFN‐1 RNA levels. (B) Secreted IFN‐1 protein levels detected in culture supernatant via HEK‐Blue SEAP reporter assay. (C) RT‐qPCR analysis of IRDS genes. Dots indicate genes known to be transcribed by u‐ISGF3. (D) Intracellular flow cytometry of IFITM1, total STAT1, and pSTAT1(y701). One representative replicate shown. Results are displayed as *n* = 3, unless otherwise noted, and presented as the mean ± SD. Statistical significance was determined using the unpaired *t*‐test. *P*‐values: *P* ≤ 0.05 (*).

Candidate genes, including IFN‐1 and those associated with IFN‐1 signaling pathways, were selected for validation. Total IFNα and IFNβ RNA were significantly higher in PDX4 CR when compared to SE (Fig. 2A). Despite the well‐known challenges of detecting of IFN‐1 [28], we chose to measure the cytokine on the protein level with a bioactive reporter system [29] which is designed to monitor IFNα/β activity via JAK/STAT activation, as measured by detection of secreted embryonic alkaline phosphatase (SEAP) under the control of ISGF3 binding. In the unstimulated state, without the presence of a DNA‐damaging agent, secreted protein expression of IFN‐1 protein was similar between SE and CR (Fig. 2B). Notably, the levels of IFN‐1 protein were close to the lower limits of detection in this assay, suggesting the capacity of cancer cells to produce IFN‐1 may be inherently restricted under basal, non‐stressed conditions.

A panel of 10 IRDS genes was selected to further validate the sequencing results. IFI6, IFITM1, BST2, and OAS1 were found to be significantly higher in PDX4 CR compared to SE (Fig. 2C). IFI6 (Interferon alpha‐inducible protein 6) is implicated in the prevention of apoptosis (via blocking the release of cytochrome C from the mitochondria), mitochondrial regulation (via stabilization of the mitochondrial membrane potential), and DNA replication [30, 31]. IFITM1 (Interferon‐Induced Transmembrane Protein 1) blocks viral entry into cells (via prevention of viral fusion), inhibits cell growth, and has been found to be required for migration, invasion, and metastasis of several cancer cell types [32, 33, 34]. Bone Marrow Stromal Antigen 2 (BST2) plays a potent antiviral role by tethering the lipid envelope of viruses to a host cell membrane and inhibiting viral release and additionally contributes to cell migration [35, 36]. OAS1 (2′‐5′‐oligoadenylate synthetase 1) binds double‐stranded RNA (typically from pathogens), activates the production of proteins that stimulate RNases to degrade viral and host RNA, and binds and stabilizes ISGs (e.g., IRF1) and AU rich elements in RNA (e.g., those in IFNβ) to stabilize and prolong RNA lifespan and IFN response [37, 38].

Permeabilized flow cytometric analysis of the cells revealed upregulation of IFITM1 and STAT1 protein in CR cells. pSTAT1, measured as a readout for JAK/STAT signaling activation, was detected with no notable changes in expression between the SE and CR (Fig. 2D, gating scheme Sup Fig. 1). These data suggest that low‐level, sustained IFN‐1 signaling, rather than robust cytokine production, may contribute to IRDS enrichment and therapy resistance.

### 3.3 Acute cisplatin exposure elicits conserved IFN‐1 signaling with distinct temporal dynamics in cisplatin‐sensitive and‐resistant cells

**Fig. 3:**
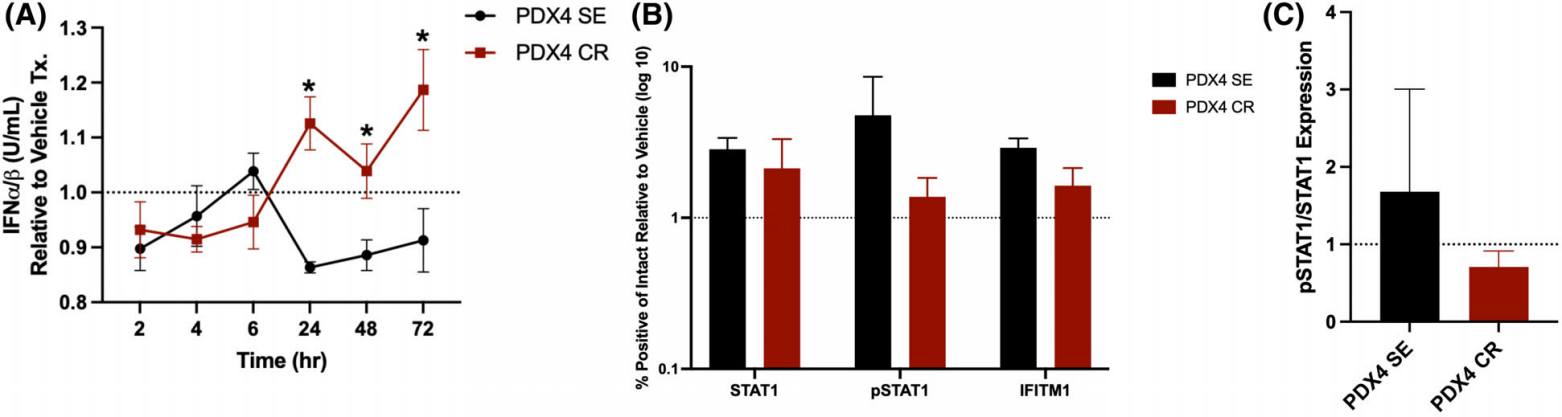
Cisplatin Resistant and Sensitive Cells Respond Differently to Acute Cisplatin Treatment. (A) Secreted IFN‐1 protein levels detected in supernatant, via HEK‐Blue reporter assay, collected at various timepoints over 72 h. Asterisks indicate un‐paired T‐test comparison between SE and CR samples at each time point indicated. (B) Flow cytometric analysis of protein expression following 48 h of IC50 cisplatin treatment. Data are normalized to untreated control cells. (C) pSTAT1/STAT1 values normalized to untreated control cells. Results are displayed as *n* = 3 and presented as the means ± SD. Statistical significance was determined using the unpaired *t*‐test. *P*‐values: *P* ≤ 0.05 (*).

To determine whether sensitive and resistant cells differ in their IFN‐1 responses to genotoxic stress, cells were treated with their respective IC50 of cisplatin for 72 h. Acute cisplatin treatment induced IFN‐1 signaling in both PDX4 SE and CR, as evidenced by an increase in secretion of IFNα/β protein (Fig. 3A). SE cells displayed an acute IFN response, wherein IFN‐1 levels increased at 6 h. However, CR cells displayed a delayed, sustained IFN‐1 response, with IFN levels increasing significantly compared to SE in the late phase of treatment. Flow cytometric analysis after 48 h of cisplatin treatment revealed near universal increases in JAK/STAT signaling activation, as well as IRDS member IFITM1 protein expression regardless of sensitivity or resistance status (Fig. 3B).

Canonical IFN‐1‐driven JAK/STAT signaling relies upon phosphorylation and dimerization of STAT1 molecules to form the functional ISGF3 complex, which transcribes genes in response to IFN‐1 signaling. However, unphosphorylated ISFG3 (u‐ISGF3) signaling is a noncanonical signaling pathway that has been found to specifically transcribe many ISGs within the IRDS gene set [20, 39]. The ratio of pSTAT1 to STAT1 protein expression was calculated and was used to indicate JAK1 kinase activity and canonical/non‐canonical signaling. Following acute cisplatin treatment, there was an observable decrease in kinase activity in CR cells, while there was an increase in SE cells, suggesting differential signaling response (Fig. 3C).

RNA was also collected at various time points following treatment. IFNβ expression was significantly decreased when PDX4 CR was treated for 72 h, as compared to vehicle treated cells. Alternatively, IFNα expression increased significantly at 24 h in both CR and SE cells (Fig. S2A). IFITM1 and STAT1 were also measured and revealed minimal changes in expression upon cisplatin treatment (Fig. S2B).

In order to identify if upregulation of the IFN‐1 related IRDS, as seen in PDX4 CR cells, was cell intrinsic or due to treatment with cisplatin, we assessed IFN‐1 and IRDS RNA levels in various other cell models, including a platinum sensitive patient‐derived cell line (PDX6), a platinum resistant patient derived cell line (PDX1), a well‐studied HGSOC cell line (OVCAR8), and a physiological ‘normal’ control cell line (FTSEC240 [fallopian tube secretory cell epithelial cell]) (Table S2). IFN‐1 and IRDS gene expression did not appear dependent on platinum resistance status and was generally considered low in all cell types observed, as expression of genes were not significantly different than the normal control cell line (Fig. S3A/B). Of interest, however, upon treatment with cisplatin, cells exhibited an upregulation of STAT1, pSTAT1, as well IFITM1 (with the exception of PDX1), indicating that IFN‐1 signaling activation in response to DNA damage may be considered a universal and conserved response. (Sup Fig. 3C).

These data indicate that an IFN‐1 response may be a conserved component of cellular response to acute DNA damage [40] in HGSOC, while prior exposure to cisplatin may alter the temporal dynamics of this response rather than its overall capacity for activation.

### 3.4 Chronic, low‐level IFN‐1 treatment phenocopies chronic cisplatin treatment

As cisplatin resistance has been associated here with sustained IFN‐1 signaling and altered temporal responses to acute genotoxic stress, we sought to directly investigate the role of IFN‐1 in the acquisition of resistance. Recent data have suggested that the role of IFN‐1 in driving pro‐tumor vs. antitumor phenotypes relies heavily on the dose and duration of the IFN signal received. On the one hand, acute and robust IFN‐1 induction appears to be a requirement for successful chemotherapy treatment [40], while chronic, low‐dose IFN‐1 activation appears to be associated with sustained or repeated DNA damage and is indirectly correlated with acquisition of prosurvival phenotypes [41]. To determine whether IFN‐1 is sufficient to drive phenotypic changes associated with therapy resistance independent of the presence of a DNA damaging agent, cells were subjected to chronic, low‐level IFNβ treatment over time. The low‐dose IFNβ regimen used to model the effects of chronic cisplatin treatment was selected following examination of IRDS and ISG expression under both high‐ and low‐dose IFNβ exposure, as well as based on measured levels of IFN‐1 secretion under both basal conditions and following cisplatin treatment (Figs 2B and 3A). Expression of CXCL10 (interferon‐gamma‐induced protein 10), a potent antiviral/anticancer protein in ovarian cancer [42, 43, 44], as well as GBP4 (Guanylate Binding Protein 4), an early IFN‐1 response gene [45], were not induced following treatment with the low‐dose IFN. However, a robust antitumor gene response was observed following high‐dose treatment, further indicating that a dual, dose‐responsive role for IFN‐1 exists and may promote differential phenotypes in a context‐dependent manner (Fig. S4).

**Fig. 4:**
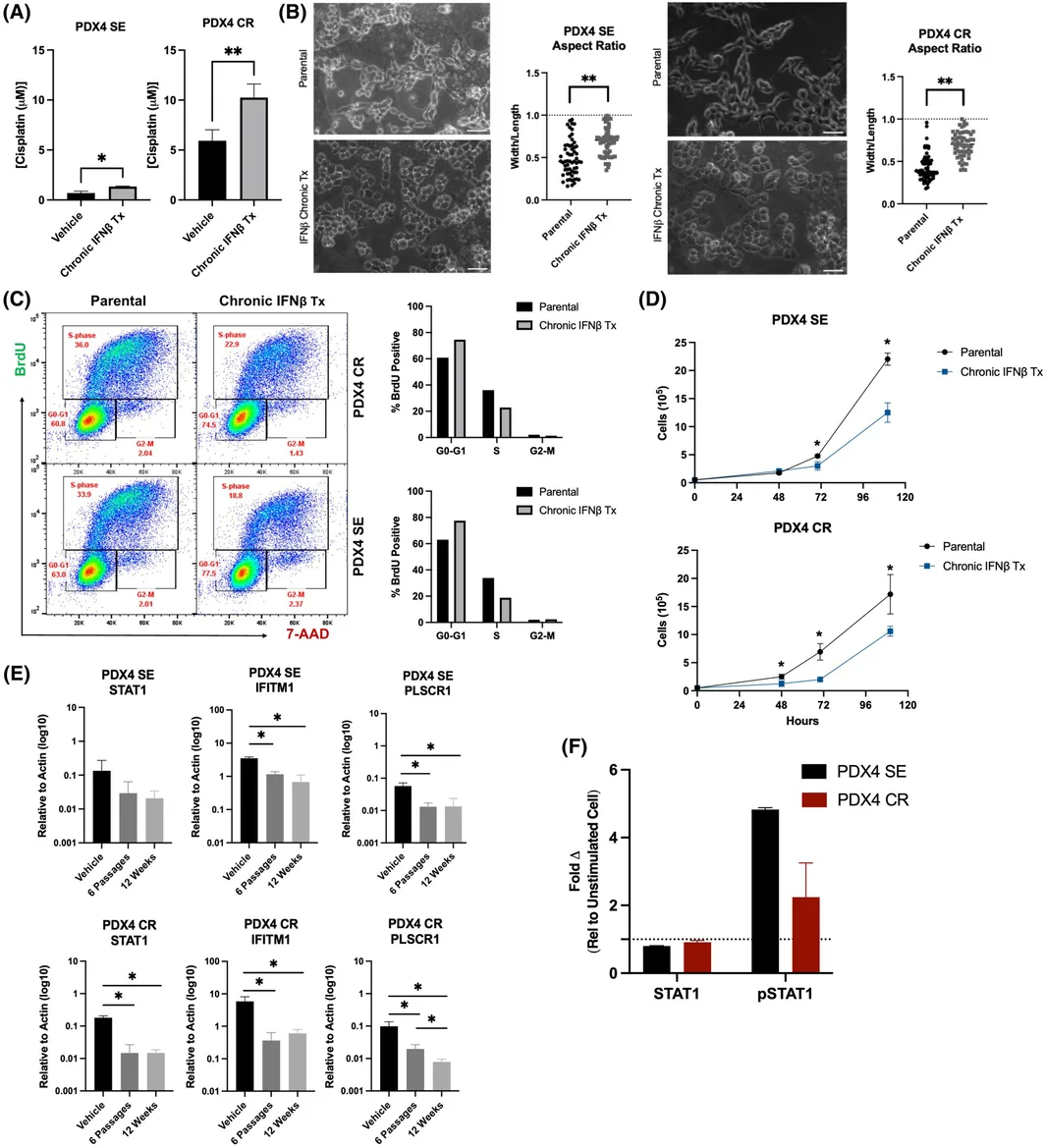
IFNβ treatment phenocopies several aspects of chronic cisplatin treatment and drives therapy resistance. (A) The IC50 of SE and CR cells was significantly higher following IFNβ treatment. (B) Morphology change was determined by measuring the aspect ratio (width/legth) of 60 cells from each sample (20 cells per photo, 2 photos per well, 3 wells). Scale bar = 50 μm. (C) BrdU staining revealed cell cycle arrest in G0‐G1 following IFNβ treatment, as measured by flow cytometry. Data represent one representative replicate. *n* = 2. (D) Proliferation was detected by counting individual wells of parental and IFNβ treated cells over time. (E) RT‐qPCR was used to analyze STAT1, IFITM1, and PLSCR1 following 6 passages and 12 weeks following consistent low‐level IFNβ treatment. (F) Intracellular flow cytometry was used to measure STAT1 and pSTAT1 protein levels following chronic IFNβ treatment. Individual replicates normalized to average control samples. Results are displayed as *n* = 3, unless otherwise noted, and presented as the mean ± SD. Statistical significance was determined using the unpaired *t*‐test. *P*‐values: *P* ≤ 0.05 (*) and *P* ≤ 0.01 (**).

Cells were treated with low‐level, 2 U·mL^−1^ IFNβ every 48–72 h, before harvesting at 12 weeks. Chronic IFNβ treatment resulted in phenotypic changes that closely mirrored those observed following prolonged cisplatin exposure. An MTT cell viability assay revealed IFNβ‐treated cells exhibited increased resistance to cisplatin (Fig. 4A). Both SE and CR cells demonstrated notable morphologic changes following treatment, exhibiting a more epithelial‐like shape (Fig. 4B). Cell cycle analysis revealed that IFNβ treatment was associated with a G0–G1 arrest (Fig. 4C). Additionally, IFNβ was found to significantly reduce proliferation in both CR and SE cells, with the SE cells specifically increasing their doubling time from 17.5 h to 22.5 h (Fig. 4D). These effects emerged progressively with continued cytokine exposure, consistent with stable cellular adaptation rather than a transient stress response. Thus, chronic and low‐level IFNβ treatment phenocopies several aspects of chronic cisplatin treatment, similar to effects noted in our previous publication [23].

Cells were harvested for analysis at both 6 passages and 12 weeks in order to monitor transcriptional changes associated with the phenotypic changes seen. Notably, chronic IFNβ exposure resulted in significant blunting of IRDS gene expression following both short‐ (6 passages) and long‐term (12 weeks) IFN treatment (Fig. 4E). On the protein level, STAT1 levels were notably decreased, while pSTAT1 levels were slightly increased (Fig. 4F). These data indicate that the cells have exerted a negative regulatory mechanism by which the response to IFNβ has been suppressed.

In order to determine whether the changes observed were due to cells having been influenced over time into an antiviral/anticancer state by IFNβ treatment, we measured the expression of CXCL10 following treatment with IFNβ and found no increases among samples treated with the low‐level dosage (Fig. S4 [42, 43, 44, 45]). We also evaluated the expression of several known negative regulatory proteins of the IFN‐1 response pathway. Interferon alpha receptor 1 (IFNAR1) expression mediates IFN‐1 responses based on availability of the receptor for ligand binding and subsequent JAK/STAT activation [46]. Ubiquitin‐specific protease 18 (USP18) is strongly induced by type 1 interferons and acts as a negative regulator of the IFN‐1 response, showing that the roles of ISGs are dynamic, multifunctional, and tunable [47]. Suppressor of cytokine signaling 1 (SOCS1) is a negative regulator of IFN‐1 signaling, acting primarily to inhibit JAK/STAT signal transduction activation via interaction with IFNAR1 (interferon alpha receptor 1)‐associated Tyk2 (tyrosine kinase 2) [48]. IFNβ did not change the expression of any of these regulators, in CR or SE, indicating other mechanisms for ISG downregulation (Fig. S5A). We then considered epigenetic remodeling as a result of low‐level IFNβ exposure. Epigenetic regulator KDM1B (lysine‐specific demethylase 1B) has been identified as an ISG that acts to mediate IFN‐1 signaling [49]. This regulator was found to be significantly upregulated in SE, but not CR, cells following treatment with IFNβ, possibly suggesting a more complicated paradigm of IFN‐1 response (Fig. S5B).

Together, these findings demonstrate that chronic, low‐level IFNβ signaling is sufficient to recapitulate key features of cisplatin resistance in HGSOC cells, establishing IFNβ as a functional driver of resistance‐associated phenotypes rather than solely a downstream consequence of DNA‐damage and genotoxic stress.

### 3.5 IFNβ‐driven resistance is observed across additional HGSOC models

**Fig. 5:**
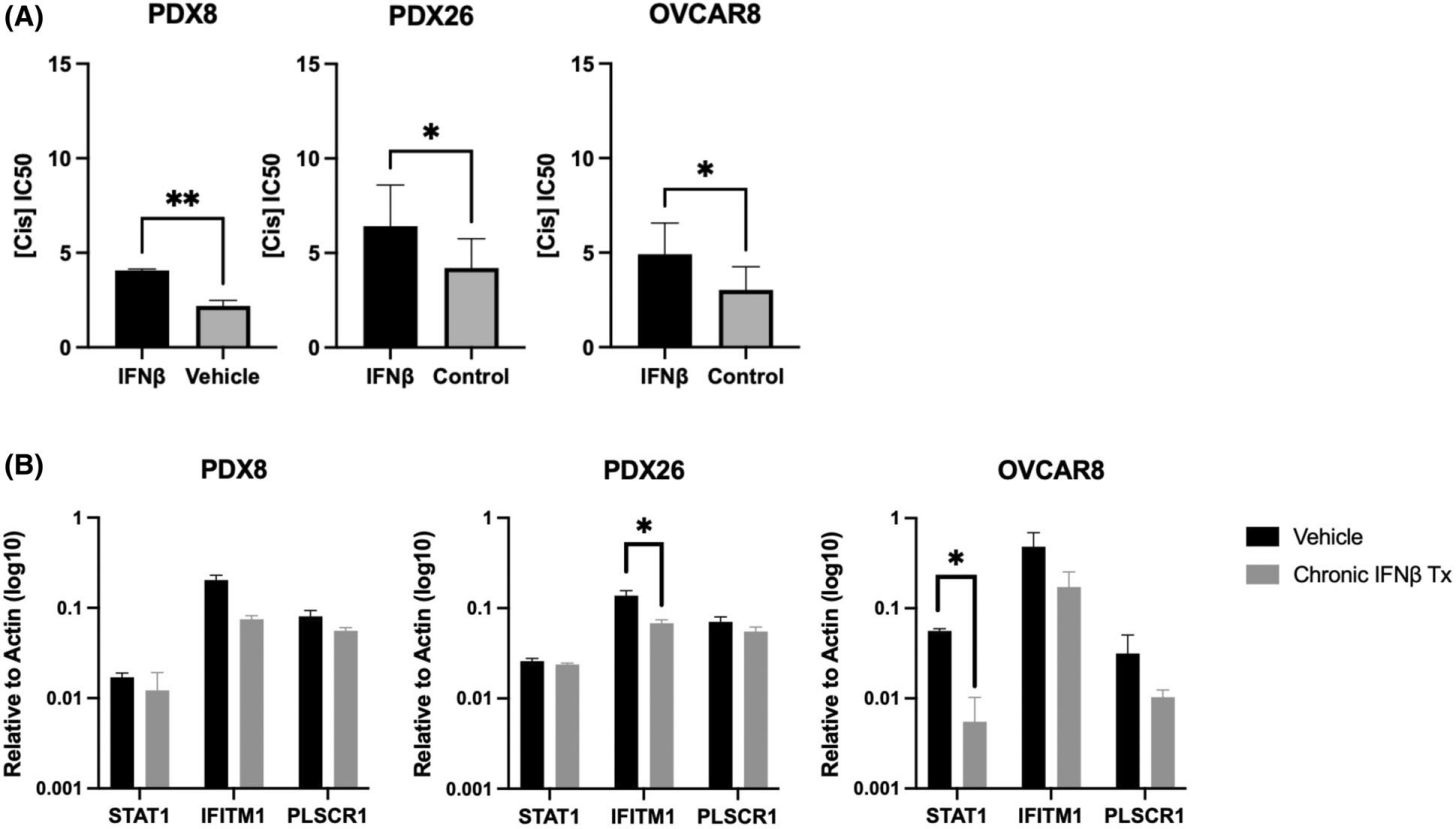
IFNβ drives therapy resistance and suppression of STAT1‐related signaling. (A) MTT cell viability assay displaying an increase in cisplatin resistance following chronic exposure to low‐level IFNβ. (B) RT‐qPCR analysis of STAT1, IFITM1 and PLSCR1 following IFN treatment. Results are displayed as mean ± SD. Statistical significance was determined using the paired *t*‐test. *P*‐values: *P* ≤ 0.05 (*) and *P* ≤ 0.01 (**).

Due to the limited nature of the paired PDX4 model, several other cell types were treated with low‐level IFNβ over time. Chronic exposure to low‐level IFNβ was associated with a significant increase in cisplatin resistance in other cell lines tested (PDX8, PDX26, and OVCAR8, Fig. 5A). The increase in resistance was also associated with trending decreases in IFN‐1 signaling related members, including STAT1, IFITM1, and PLSCR1 (Fig. 5B). These data reinforce that low‐level IFNβ is a functional driver of resistance in HGSOC, potentially via suppression of STAT1 signaling.

### 3.6 A low‐level IFN‐related signature correlates with worse survival

**Fig. 6:**
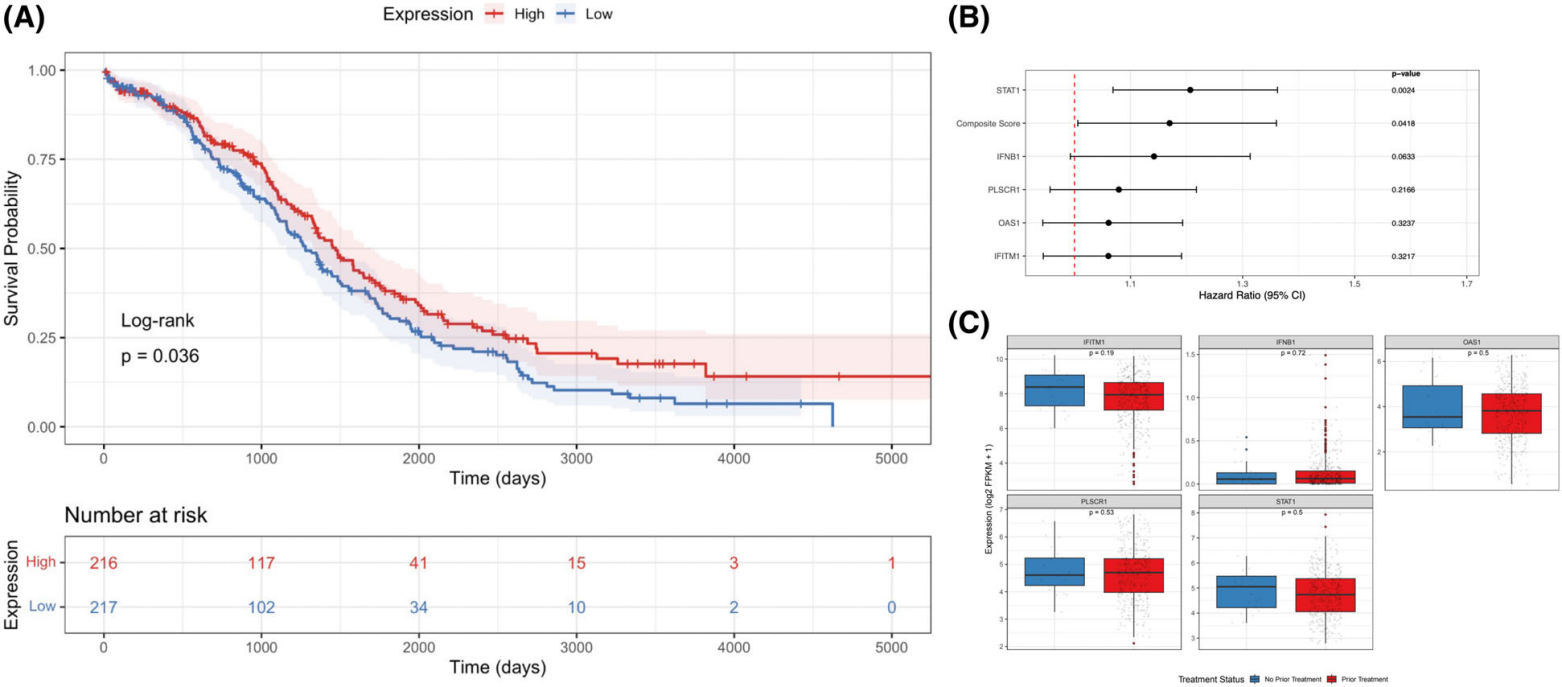
Evaluation of IFN‐1‐related gene set clinical relevance through TCGA. (A) Survival probability of patients within the TCGA ovarian cancer database based on high (red) or low (blue) expression of an IFN‐1‐related gene set. (B) Cox hazard ratio forest plot of individual genes making up the IFN‐1 gene set, as well as the composite score of all genes. The reference groups used are HIGH values of specific gene expression. (C) Analysis of individual gene expression based on history of prior therapy. Box and whisker plots show median (dark line), first and third quartile (boxes); faint dots: extreme and sparse outlier dots near the ends of the distribution; bold dots: dense outlier dots that are closely packed next to one another (overlap in color which is enhanced as bold) and close to the whisker tail boundaries.

To establish the clinical relevance of low versus high IFN‐1 related signaling, a small IFN‐1 related gene signature was defined, composed of IFNβ, STAT1, IFITM1, PLSCR1, and OAS1. Low expression of this gene set was associated with worse overall survival in the cohort (Fig. 6A). Cox hazard ratio analysis suggests that STAT1 is the main gene driving the weight of the gene set, as high expression was significantly associated with a protective role in patients (Fig. 6B). IFNβ itself exhibited a trend toward significant association, however did not reach significance, along with the other individual genes evaluated.

IRDS and IFN‐1 expressions in the PDX4 model appear to be driven by recent cisplatin treatment. Thus, the individual genes within the IFN‐1 signature were evaluated for association with prior treatment. There were no differences in the expression of these genes based on if the patient had or did not have prior therapy, suggesting that IFN‐1 activation is likely a transient response to DNA damage (Fig. 6C).

These data reinforce the theory that low IFN‐1 activation may be associated with pro‐tumor phenotypes and worse overall survival and that IRDS activation may primarily be associated with recent or chronic DNA damage and patient/tumor‐specific.

## 4 Discussion

Ovarian cancer represents a unique model of the development of therapy resistance. Most patients initially achieve partial or complete response, yet the majority ultimately recur. Recurrence following standard therapy is associated with therapy resistance and tumor aggression, indicating a causal link between treatment and recurrence [50, 51]. DNA damage, from genetic instability and/or standard therapy, is associated with activation of IFN‐1 signaling and IRDS in HGSOC. However, whether IFN‐1 plays a functional role in driving resistance and associated phenotypes or is merely a transient byproduct of chronic DNA damage, which drives resistance in specific cells remains unknown [52]. Our findings identify IFNβ as a functional driver of cisplatin resistance in HGSOC, capable of inducing phenotypic changes independent of the presence of a DNA‐damaging agent and treatment‐related IRDS upregulation.

IFN‐1 as a marker of DNA damage, and even successful chemotherapy treatment, has been observed across multiple cancer types [17, 40]. However, the precise role of IFN‐1 in driving resistance within ovarian cancer has not been well‐established. The IFN‐1 upregulation has been heavily implicated in mediating DNA damage resistance via the IRDS gene set, with few specific mechanisms identified in ovarian cancer [17, 18, 20]. Our study aimed to clarify the role of IFN‐1 in driving the phenotypes associated with therapy‐induced DNA damage, while investigating if IRDS also plays a key role. The stable IFN‐1 activation and signaling phenomenon seen in PDX4 CR, compared to the SE counterpart, appears to be exclusive to cells chronically, and perhaps recently, exposed to cisplatin. While RNA expression reflected this activation, the absence of corresponding robust protein secretion indicated low‐level, cell‐autonomous, autocrine‐like signaling. We hypothesized that chemoresistant cells, with enrichment of the IRDS program, would be ‘primed’ to respond to the stress of therapy via robust non‐canonical IFN‐1 signaling activation. However, upon acute treatment with cisplatin, an increase in STAT1, pSTAT1, and IFITM1 protein was observed in both SE and CR cells, as well as various other cell types, indicating that a conserved IFN‐1 response to DNA damage may exist.

Consistent with previous reports on the activation of IFN‐1 signatures following both chemo‐ and radiation therapies [17, 40, 53], we identify IFN‐1 and IRDS upregulation in CR cells, following chronic cisplatin exposure. The current IFN‐1/resistance paradigm asserts that DNA damage drives IFN activation and subsequent IRDS to contribute to resistance. The data presented here further defines this assertion, validating that acute DNA damage indeed drives IFN and IRDS activation, while only some cells maintain this signaling. In this case, CR was primed for non‐canonical ISG activation, while SE was not. Further investigating the role of IFN/IRDS in resistance, differential temporal upregulation of the cytokine in SE and CR cells was observed following acute cisplatin exposure, with SE displaying acute and robust IFN‐1 activation and CR responding with latent, non‐canonical IFN‐1 signaling. These data suggest that sustained IRDS programs that contribute to resistance may be cell‐intrinsic, such as within the CR cells, which prime or prepare them for differential response to genotoxic stress. TCGA data further validates this hypothesis as well, indicating that tumors do not maintain a static IFN‐1/IRDS signature following therapy exposure.

The phenotypic changes seen in both SE and CR upon chronic treatment with IFNβ are consistent with the emergence of a drug‐tolerant persister (DTP) cell phenotype. The DTP cell phenotype has been characterized as a reversible phenotype that cancer cells adopt to tolerate therapy exposure, mimicking early embryonic diapause, wherein they become slow‐cycling and rely on autophagy programs for survival [54, 55]. Following treatment, both SE and CR cells were found to arrest in G0–G1 and become both less proliferative and more resistant to cisplatin, indicating a shift into dormancy or a quiescent‐like phenotype [56]. Suppression of both canonical and non‐canonical IFN‐1 programs suggests the cells have transitioned into a stress survival state encompassing slow‐cycling, suppressed transcription to allow the cells to persist through treatment. Further, specific suppression of STAT signaling has been associated with tumor cell dormancy, via methylation of SOCS1 [57]. These data are consistent with a previously undefined role for low‐level IFNβ in shaping tumor‐cell states associated with reduced proliferation and drug tolerance, independent of IRDS activation and DNA damaging agents.

As shown, IFNβ treatment promoted the development of resistance while driving significant blunting of both canonical and noncanonical IFN‐1 signaling programs, prompting further investigation into the precise mechanism of the promotion of therapy resistance. Most notably, STAT1 expression was significantly decreased in IFNβ‐treated cells, suggesting its potential role in mediating resistance via altering signal transduction dynamics or availability. Separate and distinct negative regulators of IFN‐1 responses were tested and revealed no changes in expression, indicating the blunting seen may be an epigenetic adaptation, as evidenced by increased expression of the epigenetic regulator, KDM1B. Of interest, chromatin remodeling proteins, such as those in the KDM family, have been associated with mediating the DTP cell phenotype, and contribute to therapy resistance [58]. Analysis from the TCGA ovarian cancer cohort suggests that low expression of an IFN‐1 related signature (comprised of IFNβ, IFITM1, PLSCR1, OAS1, and STAT1) correlates with poor overall survival, and this is primarily influenced by levels of STAT1 expression. These data further indicate that STAT1 may be a clinically relevant, master regulator in driving the pressured phenotypic responses associated with low‐level IFNβ exposure. Taken together, this evidence suggests that IFN‐1 in the absence of DNA damage may contribute to resistance in an alternative, IRDS‐independent, STAT1‐dependent manner.

The current paradigm of IFN‐1 production in cancer suggests that DNA damage, driven by p53, or homologous recombination deficiency (HRD)‐associated mutations, may be sufficient to drive chronic IFN‐1 production and contribute to pro‐tumor phenotypes, as suggested here [16]. In ovarian cancer specifically, localized IFN‐1 and related signatures have been found to pre‐exist [59], being identified as early as the emergence of p53 signatures, correlating with immune exhaustion as carcinoma progresses and increasing following chemotherapy exposure [19, 60]. Further, IFN‐1 activation or suppression has been found to characterize therapy naïve and chemo‐refractory HGSOC, respectively, further suggesting that a suppressed IFN‐1 signature may promote different phenotypes and may be exploited therapeutically as observed by cisplatin sensitization when treated concurrently with high‐dose (10 000 U·mL^−1^) IFNα [61]. Of note, the precise roles of IFN‐1 in both physiologic and pathologic settings within the female reproductive tract remain quite paradoxical and require further investigation. Distinct IFN‐1 ligands appear to play various roles differentially contributing to tumor progression, immune activation, and therapeutic response depending on cellular context and signaling environment. While members such as IFNα/β have been shown to play both pro‐ and antitumor roles in cancer based on dose and duration, IFNε is generally considered to be a tumor suppressor, robustly expressed in the female‐reproductive tract, with loss consistent with tumorigenesis [62, 63]. Thus, much work needs to be completed to fully understand the extent of IFN‐1 heterogeneity in ovarian cancer.

Many preclinical and clinical studies observing and modulating the roles of IFN‐1 production and signaling are focused on monitoring immune activation and overall tumor burden, rather than elucidating the precise roles of tumor cell‐intrinsic IFN‐1 signaling [64]. We focus here on systematically determining the role of cancer cell‐autonomous IFNβ itself in promoting a pro‐tumor phenotype. The limitations of this study include the use of a single, paired patient‐derived cell line (PDX4) in modeling developed therapy resistance and IFN‐1 activation, and the *in‐vitro* model itself. Future studies will incorporate the use of additional paired sensitive and resistance cell lines, as well as 3d‐systems and animal models to provide full context of the delicate dynamics of IFN‐1 signaling, and will determine the factors that contribute to intrinsic IFN‐1 activation, as well as determine specific mechanisms by which IFNβ itself promotes resistance.

## 5 Conclusion

We demonstrate that an IFN‐1‐related signature is enriched in cisplatin‐resistant HGSOC cells following chronic cisplatin exposure. Following acute DNA damage, therapy sensitive and resistant cells demonstrate differential IFN‐1 and ISG responses, wherein although IFN‐1 induction is a universal response to DNA damage, resistant and sensitive cells display varying temporal kinetics consisting of acute and latent IFN‐1 upregulation, respectively. IFNβ was found to be a functional driver of therapy resistance, phenocopying other key features of chronic cisplatin exposure such as slow cycling/drug‐tolerant persister cell phenotype associated with a decrease in STAT1 signaling, and epithelial morphology changes, independent of IRDS activation. Analysis of the TCGA ovarian cancer database confirmed the relevance of low STAT1 and worse overall survival, further suggesting clinical relevance of sustained and chronic IFN‐1 signaling. This evidence provides a basis for the continued research into the various heterogenous and context‐dependent roles of IFN‐1 in promoting various phenotypes in HGSOC, as well as the specific mechanisms underlying chronic IFNβ related acquired resistance.

## Conflict of interest

The authors declare no conflict of interest.

## Author contributions

Conceptualization was completed by AC and JU. Data curation was performed by AC and JU. Formal analysis was completed by AC, VSSAA, GY, and JU. Funding was acquired by YI, CH, and JU. Investigation was completed by AC, TS, KM, VSSAA, SA, JD, EN, JC, and JU. Methodology was conceptualized by AC, TS, KM, VSSAA, JC, GY, CH, and JU. Project administration was overseen by AC and JU. Resources were obtained by JU and CH. Writing of the original draft was completed by AC and JU and all authors participated in final edits and review.

## Supporting information


**Table S1.** RT‐qPCR primers.
**Table S2**. HGSOC cell line characteristics.
**Fig. S1**. Example gating scheme for flow cytometry.
**Fig. S2**. Temporal expression of IFN‐1 and IRDS RNA following cisplatin treatment.
**Fig. S3**. IFN‐1 and related signature expression in other cell types.
**Fig. S4**. IFNβ dose for generating acute responses.
**Fig. S5**. Validation of IFN‐1 Signaling Regulatory Mechanisms.

## Data Availability

The data that support the findings of this study are openly available in The Cancer Genome Atlas—Ovarian Serous Cystadenocarcinoma (TCGA‐OV) at https://portal.gdc.cancer.gov, reference number phs000178. Data from Suzuki et al. [23] are contained within the article and Supporting Information.
